# Cholera Morbus

**Published:** 1820-12-01

**Authors:** 


### SPASMODIC CHOLERA OF INDIA.

1. *Report on the Epidemic Cholera Morbus, as it visited the Territories subject to the Presidency of Bengal, in the Years* 1817, 1818, *and* 1819. *Drawn up by order of the Government, under the Superintendence of the Medical Board.* By James Jameson, Assistant Surgeon and Secretary to the Board. One vol. 8vo, pp. 325. Calcutta, 1820.

2. *Account of the Spasmodic Cholera which has lately prevailed in India and other adjacent Countries and Islands, &c. in a Letter from Mr. Corbyn to Sir Gilbert Blane.* Medico-Chirurgical Transactions. Vol. XI. part 1, 1820.

"Noxia si penitus Choleram sævire venena." Ser.

We have given so full an account of this tremendous epidemic in our Review of the excellent report drawn up by the Bombay Medical Board, that we dare not trespass on the patience of our European readers by entering into an analysis of the present documents. Mr. Jameson appears to us to have drawn up a very impartial digest of the various returns made by full 100 medical officers. It could hardly be expected that no discrepancy of opinion should prevail respecting the cause and treatment of such a wide spreading epidemic. There was, in fact, considerable clash of sentiment, but as far as therapeutics were concerned, a very large and preponderating majority of evidence furnished ample grounds for the following conclusions, which we shall give in the words of the author.

1. "The disease sometimes attacked with such extreme violence, as, from the commencement, apparently to place the sufferer beyond the reach of medical aid, and to render every curative means employed equally unavailing.

2. "The difference in the degree of mortality amongst those who did, and those who did not, take medicine, was such as to leave no doubt that, when administered in time, and with discrimination, it frequently saved the patient from death.

3. "The chances of a patient's receiving benefit from medicine, diminished in proportion with the increased duration of the attack.

4. "In Europeans generally, and in robust natives, bleeding could be commonly practised, where the patient was seen within one, two, or perhaps three hours, from the beginning of the attack; and in all cases, in which it is resorted to, under such favourable circumstances, it was more successful than any other remedy in cutting short the disease: usually resolving spasm; allaying the irritability of the stomach and bowels; and removing the universal depression under which the system laboured.

5. " Amongst the generality of natives, the depressing influence of the disease was so powerful and rapid in its operation, as almost immediately to produce a complete collapse, and nearly destroy arterial action; and therefore to render venesection for the most part, from the beginning, impracticable.

7. " Although it cannot be affirmed that calomel possessed any specific power in checking the disorder, it was undoubtedly frequently useful in soothing irritability; and was, perhaps, of more certain sedative operation than any other medicine." 247.

Whether it was that the epidemic, in a few places, totally changed its nature, or that the mental telescopes of a few individuals had one lens more, or one lens less than those of the generality of mankind, (of which we see occasional examples in this country) but so it was, that the abovementioned remedial measures found useful by nine-tenths of the community, not only failed, but proved *highly prejudicial* in the hands of some.

In a supplement to the work it appears that subsequent to the month of June 1819, the disease re-appeared in the upper provinces, and, it would seem with some modification, as bile was frequently seen in the stools; and re-action was more violent. It is not difficult to conceive that, under such circumstances, " large and repeated bleedings proved the only efficacious means of opposing the disorder."

Of the *remote* causes of this epidemic, Mr. Jameson, and consequently the Calcutta Board, can offer nothing satisfactory. They conceive that it could not be owing solely to atmospherical vicissitudes—though they were great—nor to contagion—nor to any thing connected with food. They conjecture that a morbific poison or miasm, however produced, was carried along by the easterly winds, and gave origin to the epidemic. This is all the explanation we can expect in the present state of our knowlege, and on which we shall make a few remarks further on.

Mr. Jameson, in labouring to subvert the hypotheses of others, respecting the proximate cause, or rather the immediate seat of the disease, has fallen, as usual, into an hypothesis himself. He endeavours to shew that the impression of the morbific cause is not exclusively made on the skin, nor on the liver; but as far as we can gather from him, it is on the *stomach*. Now this we think is only substituting one *exclusive* doctrine for another. We believe that all the great organs of the body are so intimately linked together, not only by blood-vessels and nerves, but by sympathetic association of function, that no one can bear the onus of disease without drawing in the others to a participation. Moreover, we cannot but conclude that a cause, so generally diffused in the atmosphere as that of an epidemic must always be, will affect a number of organs and parts simultaneously—particularly the whole of the nervous or sentient system distributed over the surface of the body, the mucous membrane of the lungs, and the lining membrane of the digestive organs. It is hypothetical then to limit the primary morbid impresssion to a single organ or tissue, however that part may appear to suffer in the course of the disease. That the nervous system in this, as indeed in almost all other epidemics, suffered the first shock, we can prove from Mr. Jameson's own symptomatology of the disease.

"The irritability of stomach, and vomiting, formed a very distressing part of the disorder. They were generally *preceded* by a feeling of giddiness, and inclination to faint." And in another place, "In some rare instances, the virulence of the disease was so powerful as to prove immediately destructive of life; *as if the circulation were at once arrested*, and the vital powers wholly overwhelmed. In these cases the patient fell down as if struck by lightning, and instantly expired." 42.

Still less will the *post mortem* researches bear out our author.

"In many, especially those who died early, the stomach and intestinal canal were found full of muddy fluid, without the slightest mark of inflammation. In others, the vessels of their inner coats were turgid, sometimes highly inflamed, ulcerated, and gangrened. The liver was congested, inflamed, and darker than usual, &c." 72.

The Bengal Board corroborate the statement of the Bombay Board respecting the non-appearance of bile in the stools or in the bowels after death. "Neither in Europeans nor in natives, was any tinge of that secretion discovered in the intestinal canal."

Mr. Jameson preserves a studied silence respecting the *first* person who pointed out this peculiar feature of the Indian cholera, and who *first* practised and suggested *venesection*, the measure on which all their hopes depend.* The Bombay Board have acted differently, and have honorably adhered to the honest maxim, *suum cuique*."

* "I have not mentioned venesection, though from its instantaneous good effects in three desperate cases (of cholera morbus or mort de

Nevertheless, leaving this and theory out of the question, the work before us has impressed us with sentiments of high respect for the talents, good sense, and general impartiality of its author.

Mr. Corbyn's communication to Sir Gilbert Blane is now more than a thrice told tale—having been published substantially in the Edinburgh Medical Journal, in the Bombay reports, and in our own journal for April last. A further experience of better than a year (being brought up to September 1819, nearly as far as the Calcutta reports before us) has confirmed Mr. Corbyn's former statements relative to the treatment of this formidable epidemic.

"The outline of the treatment alluded to, is, to administer twenty grains of calomel (in powder, not in pills) and to wash it down with sixty drops of laudanum and twenty drops of oil of peppermint in two ounces of water—to bleed freely in the early stage—and to support the warmth by external heat, the hot bath and hot friction, and internally by cordials." 122.

Sir Gilbert Blane, in a commentary on the different communications, has laboured to render it at least probable that this epidemic was *contagious*. It is sufficient to say that the Calcutta Medical Board, who had better opportunities of ascertaining this point than Sir Gilbert Blane, give a decided negative to the supposition.

Sir Gilbert Blane has been favoured by the Army Medical Board, with a document from the principal medical officer in the Isle of France, shewing that the epidemic appeared there on the 20th November 1818. It has since raged with great violence.

Here, as in India, the laborious classes of the population suffered most. "With regard to the practice, opium and calomel were administered to the cases in the army, but in smaller dosess than in India." The principal medical officer denies contagion, attributing the epidemic to atmospheric influence. The inhabitants, however, believed the infection was imported by the Topaze frigate! Such popular *beliefs*, like some popular *disbeliefs* here, are little worthy of notice.

"Non ego ventosæ *plebis* suffragia venor."

---

chien) I am inclined to think that it might prove a powerful auxiliary in relieving the brain and other internal organs, when overwhelmed with blood, anterior to reaction; and also by moderating the reaction itself." *Johnson on Tropical Climates, p.* 412; *first edition,* 1813. How far the abovementioned anticipations have been realized, we leave Mr. Jameson to judge.

The inhabitants of Bourbon, acting on the contagious creed, instituted a strict quarrantine. But the epidemic laughed to scorn these little hypothetical barriers, and marched into the place without ceremony.

One of the medical officers having stated his opinion that the cause of this epidemic was owing to the issue of a morbific effluvium from the earth, as was long ago maintained by Sydenham, Sir Gilbert Blane characterizes this opinion as "an assumption purely gratuitous, and neither supported by fact nor countenanced by analogy." Now we would ask Sir Gilbert Blane if the matter of contagion, or the febrific miasm from marshy soils, has ever been rendered cognizable to the senses?—and what proof have we of their existence but by their effects? The epidemic in question, as well as many other epidemics, could not be traced to contagion, for even, according to Sir Gilbert's own confession—"it has been found occasionally, like the small-pox, to break out in spots a few (he might have said a few hundred) miles distance from the known seat of contagion, *without its being possible to trace it.*" The idea of contagion then being almost universally given up, we have but two other probable sources—the earth and the air. The longer we have reflected on this subject, the more we are convinced of the truth of Sydenham's conjecture. We know that certain states of the earth's *surface* will disengage morbific agents. But it will be triumphantly asked, "have these agents or effluvia been ever *seen* issuing from the *bowels* of the earth?" We answer, by asking if they have ever been seen descending from the regions of the air, or passing from one person to another? And are there no subterraneous agents at work? Do we never feel the earth itself tremble under our feet from one extremity of Europe to the other, from the agency of subterraneous and unseen causes? Have we not seen pestilences quickly succeed these intestinal commotions of nature? Do we not actually see the electric fluid itself, at one moment forsake the air and plunge into the bowels of the earth; while the next instant, it springs from thence to the clouds over our heads? And is morbific effluvium a *less* subtle fluid than the electric? Oh! but, says Sir Gilbert Blane, "how is it conceivable that these effluvia could exhale from the earth in the progressive manner in which this disease extended itself, and how will it account for its appearing *on board of ships at sea?*" In answer to this we must first state, that the great Eastern epidemic spread from one extremity of India to the other, often *directly against the monsoon.* Now, how is this reconcileable with atmospheric influence? It would be very curious, too, if human *contagion* had the power of selecting a single point out of the thirty-two in the compass, and of refusing to travel for a time on any other parallel! It would be equally curious if *atmospheric* influence could propagate itself directly against a trade wind, which blew in one direction for six months together!

Indeed, the capricious as well as obstinate courses which this epidemic occasionally pursued are much more explicable on the principle of a terrestrial, than of an atmospheric or contagious influence. We see the causes which produce earthquakes take the most irregular and unaccountable routes; and as for this morbific agent appearing at sea, we can have no great difficulty in conceiving the possibility of such an occurrence, after seeing, in our own days, volcanic islands boiling up from the bottom of the ocean.

Upon the whole, we think that we have been much too precipitate in rejecting the opinion of Sydenham, and that no other hypothesis, if such it be, is half so plausible as the terrestrial origin of epidemic influence, however that influence may be subsequently transported about, or modified by atmospheric constitutions.

And here we cannot help stating it as our decided conviction, that the ever-varying *causes* of epidemic diseases will produce an ever-varying character of them, and consequently an ever-varying pathology and treatment. This may be mortifying to the pride of man, who often builds an ingenious theory on the symptoms and treatment of a single epidemic, the whole foundation of which is shaken to the centre by the next visitation of disease. It is in vain to say that epidemics differ only in the organs principally affected. What produces this difference of seat? Here we must recur to an *occult* cause, however we may be inclined to account for things without it. The fact is, what all unbiassed observers have long ago acknowledged, that not only do the causes and seats of epidemic diseases materially differ at different epochs; but their whole nature is modified, and requires an ever-varying modification of management. Nor do we think that this impassable bar to perfection is at all injurious to medical science. If pathology and therapeutics could be reduced to certain fixed and invariable rules, inquiry would languish, and the human mind would soon lose its most powerful stimulus to exertion. Medicine might then be administered by the mere routinist with as much success as by the most intelligent physician. But there is no fear of this consummation in the practice of physic! Our descendants will have to go all over the same ground that we are treading, and probably not a single tenet of the present time will hold good fifty or even thirty years hence. But if we roll the stone of

Sisyphus, it is not in vain. The exertion, though it may be useless to futurity, is salutary, nay absolutely necessary for us. If our utmost efforts are incapable of placing us one step in advance, still a moment's cessation from labour would inevitably cause us to retrograde. But to return.

The Army Medical Board have recently received intelligence from Ceylon, and with their accustomed liberality have communicated the same to the profession.

Dr. Davy, who is already known to our readers, considers that the epidemic was unconnected with the direction of the winds, the topography of the places visited, or any sensible changes in the state of the atmosphere. In some cases, the flaccidity of the muscular parts after death, resembled that produced in animals by electricity, or when hunted to death. The colour of the venous and arterial blood was the same—both being of the dark venous hue. The blood drawn never presented a buffy coat. An analysis of the air expired from the lungs of the sick, did not contain more than one third of the carbonic acid contained in the breath of healthy people. Mr. Finlayson observed in some cases, what happened often in Bengal, that the operation of the morbific cause was so violent as to destroy life in a few hours, without any of the characteristic tokens of the disease, except the extreme prostration of strength. The warm bath and all other medicines seemed rather hurtful than beneficial.

"Non vota, non ars ulla correptos levant!"

In these particular cases there was such great congestion of blood in the brain "that it had the appearance of being enveloped in a layer of dark coagulated blood, or by a diffuse and general ecchymosis, and in some cases, when it was cut into, large quantities of dark coagulated blood gushed from it and from the theca of the spine." In the ordinary form of the disease, this appearance was wanting, the blood being principally collected in the abdominal viscera. "The blood was so fluid that any opening of the larger vessels produced an inconvenient effusion. In several cases, the surface of the heart and pericardium was lined with a green coloured gelatinous fluid." There was found a dark-coloured fluid in the stomach, and a colourless fluid in the rest of the intestines, which were blanched like tripe. These appearances were peculiar to cases of early death. In the more advanced stages, the morbid appearances did not differ materially from what has already been described in our eighth number. The deaths, in several of the stations, equalled the recoveries, or even exceeded that proportion. In two cases, the spasmodic contractions continued for some time after death! "The stress of the cure was laid on twenty or thirty grains of calomel given at first, and repeated in doses of eight or ten grains every second, third, or fourth hour. Blood-letting was practised with the same relief as in other parts of India." We fully coincide with Sir Gilbert Blane in the following passage.

"We cannot conclude this article without remarking that the medical officers of the British empire in India have done themselves much honour, by the great ability, zeal and humanity displayed in the preceding communications."

Our brethren in the Eastern hemisphere have had most arduous duties to fulfil during the last three years, and we have reason to believe that the manner in which they performed them has reflected credit on the profession, and on humanity.

"Vir bonus, quod honestè se facturum putaverit, faciet, etiamsi laboriosum erit:—faciet, etiamsi damnosum erit:—faciet, etiamsi periculosum erit."

